# Understanding investigators’ attitudes through a results-based accountability framework within a single CTSA institution

**DOI:** 10.1017/cts.2016.15

**Published:** 2017-01-13

**Authors:** Erin Rothwell, Trent Matheson

**Affiliations:** 1 College of Nursing, and School of Medicine in the Division of Medical Ethics and Humanities, University of Utah, Salt Lake City, UT, USA; 2 Center for Clinical and Translational Science, University of Utah, Salt Lake City, UT, USA

**Keywords:** Common metrics, Investigators, Interviews

## Abstract

**Purpose:**

The National Center for Advancing Translation Science (NCATS) is implementing a new strategic management plan called the results-based accountability framework. This framework is part of the common metrics initiative. For successful implementation and adoption of new management strategies, it is important to assess current stakeholders’ experiences and needs.

**Methods:**

Interviews were conducted with principal investigators who are conducting research and supported by the Center for Clinical Translational Science at the University of Utah. Between July and August 2016, 15 interviews were completed and audio recorded. A qualitative content analysis was conducted on the transcripts.

**Results:**

Results indicated the need to provide education about the continuum of clinical translational research; time constraints during pre-award; barriers to IRB submissions; difficulty balancing other responsibilities in academic health centers; and the need for shared study coordinator resources.

**Conclusion:**

Implementing a new management philosophy requires an understanding key stakeholders attitudes and needs. The research identified ways to help engage investigators with centralized resources supported by NCATS and implementation of common metrics at this university.

## Introduction

The purpose of this research was to identify perceptions and attitudes toward the clinical translational research processes within a single university hub funded by the Center for Translational and Scientific Award (CTSA). We used a qualitative approach and interviewed principal investigators (PIs) conducting research supported by one CTSA hub (University of Utah Clinical and Translational Science Center-CCTS). Interviews were focused on eliciting PI perceptions related to the implementation of a new strategic management plan, the results-based accountability (RBA) framework used in the common metrics initiative, from the National Center for Advancing Translation Science. As each hub within the CTSA Consortium is required to implement this new management plan, it is important to assess current stakeholders’ experiences and attitudes for maximizing successful implementation and adoption of new management strategies.

The RBA framework includes 5 domains: (1) assessment of current program performance; (2) identification of factors that contribute to or hinder progress; (3) identification of existing and potential collaborators to advance progress; (4) prioritization of the most important issues to improve progress; and (5) development and implementation of a plan to improve outcomes [1].

It is important to note that these domains are typically used to examine a particular common metric (ie, median Institutional Review Board [IRB] review time). For this study, we used the framework to assess investigator perceptions of the clinical trials processes at the University of Utah. The domains of the framework were used to develop questions contained in the semistructured guide asked during interviews. This approach both introduces individual investigators to aspects of the RBA framework and allows the CCTS to obtain feedback on PI perceptions of the clinical research processes at our institution. We hypothesize that understanding the knowledge, perceptions, and “culture” of PI stakeholders within a specific CTSA hub may be helpful for implementing this new management philosophy.

## Methods

A convenience sample of PIs who had used the CTSA in the past year were contacted for participation (n=31). Interviews were conducted with 15 investigators (48%) during July and August 2016. Interview times ranged from 10 to 30 minutes and were audio recorded. Recordings were transcribed and a qualitative content analysis was conducted using the software Dedoose 7.0.23 (Dedoose 7.023, 2016).

The demographics of the CCTS investigators interviewed are shown in Table 1.

**Table 1:** Demographics (n=15)

Sex	33% female (n=5)
Rank	47% Assistant Professors (n=7) (1 was a KL2 graduate) 20% Associate Professors (n=3) 33% Professors (n=5)
Degree	13% Ph.D.s (n=2) 87% M.D.s (n=13)
Funding type	80% both NIH and industry funding (n=12)

NIH, National Institutes of Health.

Questions were based on the RBA framework and included perceptions of the translational research process, barriers to research, resources the CCTS could provide to assist PIs, and means by which the CCTS could improve outcomes and productivity of the clinical trials process.

## Results

Six themes were identified from the transcript-based content analysis. These included: (1) improve education about the continuum of clinical translational research; (2) preaward support needs; (3) networking for private-public partnerships; (4) IRB/contract support; (5) study coordinator support; and (6) assistance with dissemination of research findings.

## Improve Education About the Continuum of Clinical Translational Research

In terms of how the CTSA could help improve the process of their clinical research, many interviewees stated they only used the CTSA as needed for specific services such as biostatistics or blood draws. No one reported the CTSA as a continuous structure that can help facilitate support over the course of the research. When asked to describe why they did not see the CTSA as a continuous resource throughout their research, investigators stated that additional responsibilities in an academic health center such as clinical care and teaching impinge on their ability to conduct research. Representative quotes include: “Consistently struggling to stay out of the clinic;” and “I use the CCTS interchangeably, because I constantly struggle with limited time and resources and clinical work.”

## Preaward Support Needs

Almost all of the participants, despite having funding, stated that there is a need for more support during the preaward process. Specific examples included the need for resources to collect preliminary data for grant applications as well as consultations on study design and biostatistics during preaward. For investigators with no funding, they stated that these challenges were magnified because of requirements to provide clinical care and teaching responsibilities within an academic health center. Thus, many interviewees stated that the CTSA could provide a “bridge” of support from preaward to postaward. For example, quotes included: “Everything is conditional until you bring in the money;” and “I mean it takes two years. [to get funding].”

## Networking for Public-Private Partnerships

About half of the interviewees discussed barriers to the creation of private-public partnerships for improving their research progress. Current involvement in industry-sponsored trials occurred as a result of direct interactions between sponsor and investigator, and was due mainly to the investigator’s access to a particular population of potential clinical trial participants. Providing a broader number of industry contacts through a CTSA would help investigators have more funding opportunities. Representative quotes include:

Usually contacted by different companies to help run different trials. It depends on how many patients you can enroll.

The PI usually has a unique relationship with different pharmaceutical companies. It is faster if we go directly.

## IRB/Contract Support

The most consistent barriers discussed from the interviewees were the difficulties of mastering the IRB language and personal opinions about the IRB process. The process of contracts through the office of sponsored projects was also associated as a barrier with the IRB. Although the participants did not complain how long the IRB review took after it was submitted, the most time-consuming process of the IRB was the actual process of the investigator completing the application. Furthermore, after an investigator learned the IRB process for submitting forms, she either borrowed language from previous applications or had the study coordinator fill out the applications and as such, was not invested in the application. Representative quotes include: “It is just boiler plate language. Plug and go. [IRB applications];” and “This is a legal document and what this does, all this does, has nothing to do with me as a researcher. It has nothing to do with the patient. This is between the hospital and its lawyers.”

The interviewees felt similarly about contracts that required substantial time and effort to construct and implement due to the uniqueness of each contract and subsequent difficulties in negotiation. Representative quotes include:

To have a single person in the research organization that does the contracts. Then that person can say to the PI, “What do you think about this fee?” “What do you think about this negotiation.” Instead we have to think about everything, there’s a lot of the red tape, a lot of publication stipulations.

Now the part that takes the longest is the negotiation and starting the whole agreement with the pharmaceutical company.

## Study Coordinator Support

Many participants also described the lack of well-trained study coordinators who can be quickly summoned to support any one clinical trial as a significant barrier. The time to train coordinators and the turn over hindered the efficiency of the clinical trials process. The most quoted needs for training of coordinators included IRB, Good Clinical Practice guidelines, and REDCap. Furthermore, many interviewees stated there are numerous instances when they need a coordinator for only a few hours per week until the next round of funding is available or for collection of preliminary data for a grant application. Most participants stated that having “bridge” support of study coordinators may provide the needed support to successfully obtain the data for the next external funding opportunity. Some quotes that capture these attitudes include:

It would be nice to have a stable pool of coordinators [in the CTSA] to use rather than hiring one individually.

Study coordinators that are supported through the CTSA. It would be great when you only need one for 10% of the time or quarter of the time.

Then the sponsor sends all the trainings. Have the coordinators done all of the trainings from the sponsors? There’s just a lot of pieces.

## Dissemination of Research Findings Support

Another significant barrier reported by many of the interviewees was the difficulty with research dissemination. This was not just in regard to writing but identification of the best mediums in which the research could be disseminated. One quote includes: “Well, that’s the big problem with investigators, is they don’t know how to disseminate findings from clinical trials.” Yet, investigators stated as the results from a research study are available that they have to spend time writing the next grant application to prevent losing protected time for research instead of publications. One quote included: “I’m too busy with my grant applications [to disseminate findings].”

## Discussion

The purpose of this study was to identify PI perceptions and attitudes toward the clinical translational research processes supported by one CTSA hub (CCTS at the University of Utah). Specifically, we explored perceptions of the translational research process, barriers to research, resources the CCTS could provide to assist PIs, and how to improve the management of the CCTS to improve outcomes and productivity of the clinical trials process. Better understanding of perceptions, and attitudes of PI stakeholders within a specific CTSA hub, should help the organization of CTSA sites to implement the new RBA management philosophy.

All the interviewees in this study stated that they tend to use the CTSA only when they need a particular service, despite the fact that the clinical translational process is not broken into individual segments. This segmentation of the CTSA services, although it appears effective for some investigators, prevents the investigator from seeing the translational impact of clinical research outside her own domain. The entirety of the translational research endeavor, ranging from planning to implementation, is important for any individual investigator to consider and understand. Furthermore, in addition to understanding the phases of translational research, it is important for investigators to consider the broader network of investigators and resources that may benefit their own research.

This philosophy of promoting use within a network is growing. As with the Trial Innovation Centers funded by the National Center for Advancing Translation Science, in which the University of Utah Hub partakes, promotes use of shared resources within a national clinical research enterprise. Engagement within a network is critical to enhance the effectiveness and efficiency of the clinical research [2]. Identifying ways to incentive investigators, especially junior investigators, to use shared clinical resources for research may address some of the barriers suggested by interviewees in this study. Our results revealed the following barriers: difficulty finding time for preaward process, IRB submissions and dissemination, difficulty balancing research desires with clinical integration, and a need for shared study coordinator resources. Thus, perhaps as individual CTSA hubs create action plans within the RBA framework that cross common metrics, it will be important to identify ways to incentivize investigators to engage with the clinical translational research process and utilize more of the CTSA. There is currently low awareness about both the common metrics initiative and the role CTSAs can play to help investigators. This study identified a few ways to reach out and help support investigators that should result in increased awareness about shared resources of CTSAs.

**Fig. 1:**
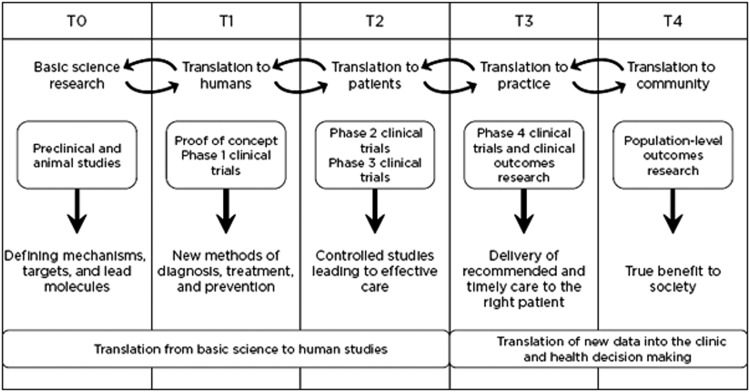
The Center for Translational and Scientific Award program at National Institutes of Health: Opportunities for advancing clinical and translational research (IOM Report June 25, 2013).

There are limitations with this study, the most notable of which is the small number of participants within a single CTSA hub and the use of only one hub. Each CTSA hub has a particular psychosocial context so these results may not be generalizable to other academic health centers. However, despite these limitations, the frequency of similar responses across the interviewees is notable. As the CTSA program moves into a new era of common metrics to improve the clinical translational research process, identifying ways to engage investigators with centralized resources can better support this upcoming generation of investigators. This can be achieved by continually engaging investigators across the clinical translational process to provide support that is institutionally specific but also similar to other investigator needs within academic health centers (Fig. 1).
